# The Medicinal Mountebank

**Published:** 1909-02-06

**Authors:** 


### Medical Antiquities.

### THE MEDICINAL MOUNTEBANK.

The quack has ever prospered, and only by the enlightenment of education will his career come to an end. Indeed, he often achieves greater financial success than those of us who have given time, energy, and money to fit us for our work. One equality with us he can rightly claim: his profession is as old as that of medicine. The masses of all ages and of all nations have been susceptible to the unscrupulous balderdash of the mendacious mountebank. The word "mountebank" is derived from the Italian "monta in banco"—mounting a bench—its application to the particular individual being due to his practice of standing upon some form of platform whilst pursuing his vocation.

The mountebank flourished until the beginning of the nineteenth century, and then began to decline in popularity. He was accompanied by a Merry Andrew, who provided the entertainment necessary to collect an audience. Their success often warranted the up-keep of some style, and it was no uncommon thing for them to travel about the country in extravagant equipages, accompanied by their clown and servants, who were often negroes or Asiatics.

Although they have passed away, their prototype still exists in the rural parts of England. The writer himself has often seen the vendor of various cure-alls standing on a chair at a country fair, exclaiming, with no small eloquence and humour, upon the excellence of his nostrum. In the market at Walsall there was, only three years ago, such a purveyor of various infallible remedies, including, unfortunately, a pill—guaranteed to contain apiol, steel, and pennyroyal—to correct all female irregularities.

At another weekly market in the North of England a muscular woman extracted teeth under an ingenious and doubtful anæsthetic. Her plan was to apply the forceps, warn the patient, and extract the tooth under cover of the noise of two huge drums placed close on either side, violently banged at the crucial moment. Her career came to a temporary close by a sentence of imprisonment, arising out of a charge for assault and malpractice brought by a victim whose mandible she fractured.

The eloquence and address of the mountebank are amusing and instructive. Jonson, in his comedy "Volpone," put into the mouth of a mountebank the following words:—

"You all know, honourable gentlemen, I never valued this ampulla, or vial, at less than eight crowns; but for this time I am content to be deprived of it for six: six crowns is the price and less in courtesy I know you cannot offer me. Take it or leave it, however, both it and I am at your service! Well! I am in a humour at this time to make a present of the small quantity my coffer contains: to the rich in courtesy, and to the poor for God's sake. Wherefore, now mark: I asked you six crowns, and six crowns at other times you have paid me; you shall not give me six crowns, nor five, nor four, nor three, nor two, nor one, nor half a ducat. Sixpence it will cost you; expect no other price, for I will not bate."

Again, an actor named Haines, well known in his day, appeared upon the new stage of charlatanage as "Watho van J. Claturbank, High German Doctor." His testimonial read as follows:—

"Having studied Galen, Hypocrates, Albumazar, and Paracelsus, I am now become the Esculapius of the age; having been educated at twelve universities, and travelled through fifty-two Kingdoms, and been counsellor to the counsellors of several monarchs. By the earnest prayers and entreaties of several lords, earls, dukes, and honourable personages, I have been at last prevailed upon to oblige the world with this notice, that all persons, old or young, blind or lame, deaf and dumb, curable or incurable, may know where to repair for cure, in all cephalalgias, paralytic paroxysms, palpitations of the pericardium, empyemas, syncopes, and naisieties; arising either from a plethory or a cachochymy, vertiguious vapours, hydrocephalous dysenteries, odontalgic or podagrical inflammations, and the entire legion of lethiferous distempers. . . . This is Nature's palladium, health's magazine; it works seven manner of ways, as Nature requires, for it scorns to be confined to any particular mode of operation; so that it affecteth the cure either hypnotically, hydrotically, cathartically, poppismatically, pneumatically, or synedochically; it mundifies the hypogastrium, extinguishes all supernatural fermentations and ebullitions, and, in fine, annihilates all nostro-trophical morbific ideas of the whole corporeal compages. A drachm of it is worth a bushel of March dust; for, if a man chance to have his brains beat out, or his head dropped off, two drops—I say two drops! gentlemen—seasonably (*sic*) applied, will recall the fleeting spirit, re-enthrone the deposed archeus, cement the discontinuity of the parts, and in six minutes restore the lifeless trunk to all its pristine functions, vital, natural, and animal; so that this, believe me, gentlemen, is the only sovereign remedy in the world. Venienti occurrite morbo—Down with your dust. Principiis obsta—No cure, no money. Quærenda pecunia primum—Be not sick too late."

Such gentlemen, who are as free with the truth as with their Latin, have passed from us. The public have in some measure been educated out of a belief in invincible cure-alls in the shape of liquid medications. We have still with us the charlatan who parades upon the music-hall stage, assisted by various manifestations of electricity, and who calls himself "the bloodless surgeon," "the electric physician," or "the iron doctor."

Electricity is a mystery to most, rumours of its therapeutic value reach the masses through the halfpenny press; and so the game goes on, the clever rogue imposing upon the ignorance of the suffering public. These tricks, lies, and quackeries work out their own end; experience teaches, education enlightens, and slowly but surely the charlatan sinks into disrepute. For a time he works in the limelight and gets some measure of fame. Our consolation is that since he has built upon the sand he must, and does, crumble and disappear.

